# Identification and Characterization of Potential Therapeutic Candidates in Emerging Human Pathogen *Mycobacterium abscessus*: A Novel Hierarchical *In Silico* Approach

**DOI:** 10.1371/journal.pone.0059126

**Published:** 2013-03-19

**Authors:** Buvaneswari Shanmugham, Archana Pan

**Affiliations:** Centre for Bioinformatics, School of Life Sciences, Pondicherry University, Pondicherry, India; Hopital Raymond Poincare - Universite Versailles St. Quentin, France

## Abstract

*Mycobacterium abscessus*, a non-tuberculous rapidly growing mycobacterium, is recognized as an emerging human pathogen causing a variety of infections ranging from skin and soft tissue infections to severe pulmonary infections. Lack of an optimal treatment regimen and emergence of multi-drug resistance in clinical isolates necessitate the development of better/new drugs against this pathogen. The present study aims at identification and qualitative characterization of promising drug targets in *M. abscessus* using a novel hierarchical *in silico* approach, encompassing three phases of analyses. In phase I, five sets of proteins were mined through chokepoint, plasmid, pathway, virulence factors, and resistance genes and protein network analysis. These were filtered in phase II, in order to find out promising drug target candidates through subtractive channel of analysis. The analysis resulted in 40 therapeutic candidates which are likely to be essential for the survival of the pathogen and non-homologous to host, human anti-targets, and gut flora. Many of the identified targets were found to be involved in different metabolisms (*viz*., amino acid, energy, carbohydrate, fatty acid, and nucleotide), xenobiotics degradation, and bacterial pathogenicity. Finally, in phase III, the candidate targets were qualitatively characterized through cellular localization, broad spectrum, interactome, functionality, and druggability analysis. The study explained their subcellular location identifying drug/vaccine targets, possibility of being broad spectrum target candidate, functional association with metabolically interacting proteins, cellular function (if hypothetical), and finally, druggable property. Outcome of the present study could facilitate the identification of novel antibacterial agents for better treatment of *M. abscesses* infections.

## Introduction

Mycobacterium abscessus, a rapidly growing mycobacterium (RGM), is the etiological agent of a wide spectrum of infections in humans. It is an acid-fast Gram-positive aerobic bacterium characterized by the presence of outer membrane which generates visible colonies within seven days of inoculation [1]. M. abscessus, being an intracellular pathogen, is responsible for severe persistent pulmonary infections, disseminated cutaneous diseases, post-traumatic, and post-surgical wound infections, mostly in immunocompetent and cystic fibrosis (CF) patients [1], [2]. In Korea and United states, M. abscessus is considered as the second and third most common non-tuberculous mycobacterial respiratory pathogen, respectively which is accountable for approximately 80% of pulmonary infections caused by RGM [3], [4]. This neglected pathogen causes a higher fatality rate compared to other RGMs and the infection of CF patients is becoming a major health-related issue in most cystic fibrosis centers worldwide [1], [2]. Several outbreaks of M. abscessus skin and soft tissue infections, following the use of contaminated medical instruments like needles or scalpels, and after surgery have been reported since 2004 [5], [6]. The pathogen also has potential to cross the blood-brain barrier causing meningitis and meningoencephalitis in immunocompromised patients [1]. American Thoracic Society (ATS) has recommended different groups of antimicrobial agents, namely, macrolides (clarithromycin and azithromycin), aminoglycosides (amikacin), cephamycins (cefoxitin), carbapenems (imipenem), glycylcyclines (tigecycline), oxazolidinones (linezolid), and quinolones (moxifloxacin) for treatment of M. abscessus infections [7]. The patients with severe infections are generally treated with long courses of combinatorial antibiotic therapy which is often accompanied by surgical resection [8], [9]. However, the emerging pathogen is not uniformly susceptible to the currently used antibiotics which varies depending on the clinical isolates. As a consequence, an optimal regimen to cure the M. abscessus infections has not been yet established [3].

*M. abscessus* is regarded as the most chemotherapy-resistant species among rapidly growing mycobacteria. The pathogen has acquired resistance to several antibiotics through mutation of genes as well as horizontal transfer of resistance causing genes [6]. Indeed, *M. abscessus* is uniformly resistant to first-line anti-tuberculosis drugs, macrolide-based (clarithromycin and azithromycin) chemotherapy, and other antimycobacterial agents, such as tetracyclines, fluoroquinolones, and sulphonamide [1], [2], [10]. Due to its intrinsic and acquired resistance to commonly used antibiotics, treatment becomes more complicated thereby leading to high failure rate [11]. Absence of an optimal treatment regimen and emergence of multi-drug resistance in *M. abscessus* stress the need for the discovery of better/new drugs to combat these infections. One of the key steps of drug discovery process is to identify novel drug targets. To this end, the present study aims to identify promising drug targets in *M. abscessus* ATCC 19977 using a systematic hierarchical *in silico* approach which can be implemented in other pathogenic organisms.

Traditional drug discovery and development process involve laborious, time consuming, and expensive experiments, often resulting a very few drug targets. In contrast, computational approach, which has become an alternative attractive way to identify all potential drug targets, could accelerate the drug discovery process, increase treatment options, and reduce drug failure rate in the later phase of clinical trials. Consequently, the utilization of computational approaches coupled with ‘omics’ data (*viz*., genomics, proteomics, and metabolomics) for searching promising drug targets has been increasing significantly in the field of drug research. The availability of complete genome sequences of several disease causing organisms and human in public databases is greatly facilitating the search of novel targets. Current computational target discovery approaches include identification of pathogen-specific essential genes, host-pathogen interaction factors, proteins involved in persistence, chokepoint enzymes, resistance genes/resistance-associated proteins; characterization of pathogen-specific metabolic pathways; prediction of gene expression levels *etc*. [12]–[17]. The approaches have been efficiently utilized to identify novel drug target candidates in several life-threatening pathogens, including *Mycobacterium tuberculosis* [14], [18], [19], *Mycobacterium leprae* [20], *Mycobacterium ulcerans* [21], *Helicobacter pylori* [22], *Streptococcus pneumoniae* [23], *Yersinia pestis* [24], and *Pseudomonas aeruginosa* [25]. Most of these target discovery methods consider selectivity/specificity and essentiality as the principal selection criteria for prioritizing therapeutic candidates. An ideal drug target must be specific to the pathogen for avoiding unwanted host-drug interactions and should be a crucial protein for growth and survival of the pathogen as inhibition of such proteins would lead to the death of the pathogen. The present manuscript introduces a novel hierarchical *in silico* approach, which integrates various computational methods, with the objective of identification and qualitative characterization of therapeutic candidates in *M. abscessus*. The current approach enables us to identify 40 promising drug target candidates, based on the criteria of essentiality and selectivity. The characterization of the candidate targets predicts their location in bacterial cell, capability to act as a broad spectrum target, functional association with metabolically interacting proteins, cellular function (if hypothetical), and druggable property.

## Materials and Methods

**Figure 1:**
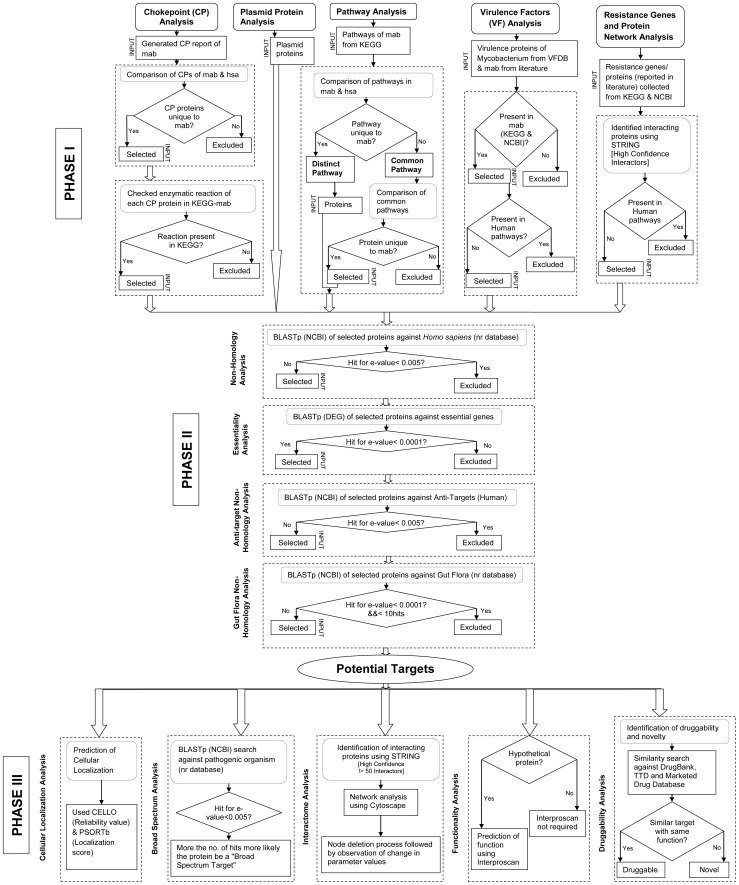
Flowchart representing the hierarchy of analyses in the study. The complete workflow illustrates each step and the selection condition followed in the three phases of analyses for identification and characterization of promising drug targets in *M. abscessus*.

A novel hierarchical *in silico* approach comprising three phases is introduced in the present study to identify and characterize potential drug targets in *M. abscessus* ATCC 19977. In phase I, the protein datasets following five different analyses, namely, chokepoint, plasmid, pathway, virulence factors, and resistance genes and protein network analysis were collected. These protein datasets were filtered through subtractive channel of analysis in phase II. In phase III, the final list of potential drug targets resulted from phase I and II was qualitatively characterized. Three phases of analyses used for screening and qualitative characterization of the potential candidate targets are discussed below. The complete workflow (Figure 1) represents various analyses and selection conditions followed in the three phases.

### Phase I: Mining of Protein Datasets

#### Chokepoint analysis

Chokepoint (CP) analysis in the metabolic network of *M. abscessus* ATCC 19977 was performed to identify unique chokepoint proteins. In the metabolic network of an organism, a reaction that either solely consumes a distinct substrate or uniquely produces a distinct product is referred as chokepoint reaction and the enzymes involved in catalyzing such reactions are named as chokepoint enzymes [26]. Blocking the action of CP enzymes, which catalyze producing or consuming chokepoint reactions, can definitely result in paucity of that specific product or accumulation of that specific substrate [15]. Chokepoint report of *M. abscessus* and human host was generated using Pathway Tools 14.5, available from SRI International [27], followed by two steps of manual curation. In first step (Figure 1), a comparative study was performed between the chokepoint proteins of pathogen (alpha (α) list) and host in order to select chokepoint proteins unique to *M. abscessus* (α1 list). The significance of this step is to avoid adverse effects on the human host, as the potential drug may also target host’s chokepoint enzymes. In the second step (Figure 1), the chokepoint proteins (α1 list) were filtered based on the presence of their corresponding enzymatic reactions in Kyoto Encyclopedia of Genes and Genomes (KEGG) database [28] resulting in α2 list. The sequences corresponding to α2 list enzymes were retrieved from KEGG database and passed through the subtractive channel of analysis.

#### Plasmid protein analysis

*M. abscessus* ATCC 19977 harbors a single circular 23 kb mercury resistance plasmid that possesses 21 protein coding genes [6]. It is evident from literature that due to the presence of *merB* within the *mer* operon, the plasmid may confer resistance to a wide range of organomercury compounds [6]. This indicates that the plasmid proteins may also act as potential drug targets. The genes and corresponding protein sequences (beta (β) list) of *M. abscessus* ATCC 19977 plasmid (Accession: NC_010394.1) were retrieved from NCBI-Nucleotide database [29] and passed through a series of subtractive analysis.

#### Pathway analysis

Pathways of *M. abscessus* ATCC 19977 were categorized as distinct and common with reference to human pathways. Pathway records of both *M. abscessus* and *Homo sapiens* (gamma (γ) list) were collected from KEGG pathway database [28]. In KEGG, pathways involved in various processes are grouped under different sections, such as metabolism, genetic information processing, environmental information processing, and human diseases. A comparative metabolic pathway analysis between pathogen and host was performed to identify the pathways that are uniquely present in pathogen (distinct pathways) and those pathways present in both host and pathogen (common pathways) [18] (Figure 1). All the proteins involved in distinct pathways and unique proteins from common pathways (γ1 list) were collected manually. The latter process of subtraction was made to avoid cross reactivity of the drug compounds with host proteins. The corresponding amino acid sequences of the selected proteins (γ1 list) from pathways were retrieved from KEGG and passed through the further subtractive channel of analysis.

#### Virulence factors analysis

Virulence factors (VF) are characterized as potential targets for developing drugs. Inhibition of such virulence proteins would lead the pathogen avirulent as these proteins are the vital cause for establishment and severity of infection [30]. Virulence Factor DataBase (VFDB) comprises virulence factors of 24 genera of pathogenic bacteria, including *Chlamydia* and *Mycoplasma* [31]. A comparison of pathogenomic composition of 24 different virulent species and types of *Mycobacterium* genus are reported in VFDB. Entire list of virulence factors and the corresponding genes were collected from the comparison table of VFDB. In addition, mycobacterial and non-mycobacterial virulence factors having orthologs in *M. abscessus* genome, reported in literature [6], were also considered for the analysis. Virulence proteins from VFDB [31] and literature [6] together constituted the delta (δ) list. The delta (δ) list of virulence proteins that are present in *M. abscessus* were retrieved from KEGG/NCBI database resulting δ1 list (Figure 1). These proteins were then examined for their presence in human pathways manually and proteins absent in the host alone were selected (δ2 list) for further subtractive channel of analysis.

#### Resistance genes and protein network analysis

Treatments of *M. abscessus* infections become more complicated as it is acquiring resistance to the existing antibiotics. The pathogen is uniformly resistant to the first-line drugs of *M. tuberculosis*, namely, rifampin (RIF), isoniazid (INH), and streptomycin (STR) [1]. It has also gained resistance to macrolide-based agents, including clarithromycin [10], a drug widely used for treating *M. abscessus* infections in last decade [32]. A novel horizontally acquired gene *erm*(41) confers resistance to macrolide-based chemotherapy [10]. The genes involved in causing resistance to standard anti-tuberculous agents were identified in some *M. tuberculosis* isolates using reverse line blot hybridization (RLBH) assay and reported in literature [33]. Mutation in *inhA* and *katG* genes causes isoniazid resistance, whereas mutation in *rpoβ* gene is responsible for rifampin resistance. Also, mutation in *rrs* and *rpsL* genes confers streptomycin resistance [33]. The orthologs of these resistance causing gene products were identified in *M. abscessus* and the corresponding protein sequences were retrieved from NCBI. Using STRING 9.0 [34], high confidence interactors for each resistance causing protein in *M. abscessus* were predicted. The resistance causing proteins and their interactors together constituted epsilon (ε) list and used in the analysis (Figure 1). The ε list proteins that are absent in human pathways were selected (ε1 list) and the corresponding sequences were retrieved from STRING database [34]. These ε1 list proteins were considered as input for the next phase (subtractive channel of analysis).

### Phase II: Subtractive Channel of Analysis

Protein datasets collected after following five different analyses (as explained in phase I) were further explored and short-listed by passing through a series of subtractive analysis. This process lead to the identification of highly selective and efficient drug targets in *M. abscessus* under study.

#### Non-homology analysis

The aim of the non-homology analysis is to identify pathogen specific-proteins that are non-homologous to the host. The significance of this step is to minimize undesirable cross reactivity of the drug and thereby to prevent its binding to the active sites of the homologous proteins in host [22]. All the short-listed proteins resulted from phase I were subjected to protein BLAST (BLASTp) search [35] against non-redundant database of *H. sapiens* with an expected threshold value of 0.005 [18], [22]. Proteins that are non-homologous to human (phi (φ) list) i.e. proteins showing no hits for the above mentioned e-value were selected for the next step (essentiality analysis).

#### Essentiality analysis

Non-homologous proteins in φ list were screened in order to identify essential proteins by a BLASTp search [35] against Database of Essential Genes (DEG) [36]. DEG 6.1, a repository of genes indispensable for the existence of an organism, contains 10,618 essential genes from prokaryotic and eukaryotic organisms. Protein alignments with less than expect value of 0.0001 [12], [24] were considered as more significant hits. Such proteins in the φ list were regarded as essential, based on the assumption that similar proteins that are essential in one organism are likely to be essential in another. The resulting proteins constituted the chi (χ) list and served as input for the next step in the analysis.

#### Anti-target non-homology analysis

Drug compounds designed to bind and inhibit the action of pathogen protein may dock with some crucial host proteins, leading to severe pharmacological effects. Such essential host proteins are termed as ‘anti-targets’, which include the human ether-a-go-go related gene (hERG), the pregnane X receptor (PXR), constitutive androstane receptor (CAR), and P-glycoprotein (P-gp). Anti-targets also include some membrane receptors, such as adrenergic α_1a_, dopaminergic D2, serotonergic 5-HT_2C_, and the muscarinic M1 [37]. The protein sequences of the anti-targets reported in literature [14] were collected. The list of anti-targets was modified by adding some new versions of sequences and removing 90 repeated/obsolete proteins which resulted in 216 anti-targets (Text S1). The short-listed sequences in χ list were compared with the anti-targets using BLASTp [35] with an e-value threshold of 0.005 [18], [22]. The χ list proteins showing no homologs in the analysis constituted the psi (ψ) list.

#### Gut flora non-homology analysis

The delineated proteins (ψ list) from the previous step were analyzed to look for their similarity with the proteome of human gut microflora. About 10^14^ microorganisms are reported to reside in the gastrointestinal tract of a normal healthy human [38]. With a symbiotic relationship, the gut microbiota play a vital role in metabolism by fermenting indigestible food particles as well as in protection from colonization of pathogenic bacteria in gut [39]. Unintentional blocking of the gut flora proteins may deteriorate the microbiota causing adverse effects in host. To avoid such circumstances, the proteins in ψ list were subjected to homology search against each of the gut flora proteome using BLASTp [35] with 0.0001 [14] as e-value threshold. The list of gut microflora reported in literature [14] was used in the analysis (Text S2). The ψ list proteins showing not more than 10 hits alone were selected and framed as omega (ω) list.

**Figure 2:**
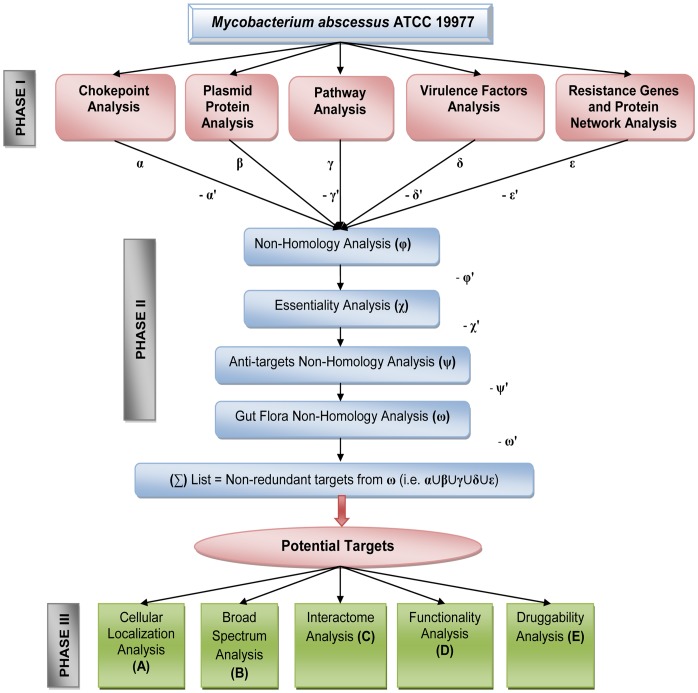
Outline sketch of the whole work. The flowchart depicts the overview of various analyses employed in the study to identify and characterize potential drug targets. Phase I starts with the collection of input proteins from five different analyses: chokepoint analysis, plasmid protein analysis, pathway analysis, virulence factors analysis, and resistance genes and protein network analysis. Proteins that enters phase II were specified as α, β, γ, δ, and ε and proteins failed in phase I as α′, γ′, δ′, and ε′. Four sequence level homology search analyses against human proteome, essential gene database, human anti-targets, and gut flora proteome were performed. The proteins passed through the analysis were represented as φ, χ, ψ, and ω and those failed were indicated as φ′, χ′, ψ′, and ω′. The short-listed targets in ω list (resulted from α&β&γ&δ&ε) were combined to form a non-redundant list of potential targets (∑ list). In phase III, the ∑ list targets (i.e. α∪β∪γ∪δ∪ε) were further characterized through cellular localization, broad spectrum, interactome, functionality, and druggability analysis (designated as A, B, C, D, and E).

The flowchart (Figure 2) depicts the overview of the three phases of analyses carried out in this study. The α, β, γ, δ, and ε input proteins resulted from phase I and II analyses were combined to form the final list of potential targets, coined as sigma (∑) list (Figure 2). The potential target proteins from *M. abscessus* ATCC 19977 were further characterized using various analyses in the next phase.

### PHASE III: Qualitative Characterization of the Short-listed Targets

#### Cellular localization analysis (A)

Microbes belonging to the genus *Mycobacterium* are categorized as Gram-positive with an outer membrane. Thus the proteins can be present in five feasible subcellular locations, namely, cytoplasm, plasma membrane, periplasm, outer membrane, and extracellular. The significance of the localization analysis is to characterize the protein as drug or vaccine target. Cytoplasmic proteins can act as possible drug targets, while surface membrane proteins can be used as vaccine targets [12]. Subcellular location information of some proteins is found in protein databases like UniProt [40]. In the absence of experimental information, subcellular localization prediction tools such as PSORTb [41] and CELLO [42] can be used. In the current study, locations of the short-listed proteins in ∑ list were identified using PSORTb 3.0.2 and CELLO 2.0. PSORTb sorts proteins by means of various modules like SVM, S-TMHMM, and SCL-BLAST using about 11692 proteins of known localization from bacteria (Gram-positive and negative) and archaea as training set [41]. CELLO 2.0 is based on two-level support vector machine system, which comprises 1444 and 7589 protein sequences as benchmark datasets for the prediction of bacterial and eukaryotic protein localization, respectively [42]. Both the tools accept protein sequences in FASTA format and gram stain option is chosen as ‘gram-positive’ in CELLO and ‘advanced gram stain’ (positive with outer membrane) in case of PSORTb.

#### Broad spectrum analysis (B)

Proteins in ∑ list were analyzed using BLASTp search [35] against a wide-range of pathogenic bacteria with an expected threshold value of 0.005 for the identification of broad spectrum targets. A list of pathogenic bacteria reported in literature [14] was considered in this analysis. In addition, several clinically important non-tuberculous mycobacteria from literature [7], [33] were included to the pathogen list. A total of 240 disease-causing bacteria from different genus were used in the broad spectrum analysis (Text S3). From the homology analysis against each of the pathogen it is theorized that close homologs present in more number of pathogens are more likely to be a ‘promising broad spectrum target’ [14]. The short-listed targets were also subjected to Cluster of Orthologous Groups of proteins (COG) search to identify homologs in other pathogenic bacteria using COGnitor [43] from NCBI which compares the query sequence with the COG database.

#### Interactome analysis (C)

A protein-protein interaction network was constructed for each of the short-listed targets in ∑ list using STRING 9.0 [34]. Protein association knowledge of STRING is derived from experimental data, gene-based analysis (neighborhood, gene fusion, co-occurrence, and co-expression), various protein interactions, and curated pathway databases [44]. High confidence interactors with score greater than or equal to 0.700 alone were included in the protein network [14]. To avoid false positives and false negatives, all interactors with low as well as medium confidence score were eliminated from the network. The interaction network of each of the ∑ list targets was analyzed using Cytoscape 2.8.1 [45], a package for biological network visualization and analysis. By the process of ‘node deletion’ each of the node in the network was deleted and characteristic changes in the values of essential parameters like clustering coefficient, characteristics path length, degree of the network, and betweenness centrality [46]–[48] were examined. The potentiality of the ∑ list targets was determined based on the change in the critical network parameter values. The importance of the query protein in the bacterial metabolic system was also determined from the number of interacting proteins (nodes) and interactions (edges) disrupted on its deletion [26].

#### Functionality analysis (D)

The function of the hypothetical proteins from ∑ list of potential targets was predicted [16] using INTERPROSCAN [49], a tool that integrates various protein signature recognition methods and databases.

#### Druggability analysis (E)

A ‘druggable’ target should have potential to bind to the drug-like molecules with high affinity. In the present study, each of the targets in ∑ list was subjected to a homology search against DrugBank 3.0 target collection [50] with an expectation value of 10^−5^. DrugBank, a large collection of drugs with the target information, includes experimental and FDA-approved drugs (6816), 4326 drug targets and 169 drug enzymes/carriers [50]. In addition, database of enzyme targets for marketed drugs [51] and the widely used drug target database Therapeutic Target Database (TTD) [52] were utilized for the druggability search. Database of enzyme targets for marketed drugs [51] comprises 71 enzyme targets from human (41), bacteria (13), fungal (4), and virus (5) with their active drugs. TTD presents 2025 targets that are classified as successful potential targets (364), clinical trial targets (286), targets discontinued from clinical trial (44), and research targets (1331) [53]. In DrugBank and TTD, each of the ∑ list targets was explored for similar therapeutic targets with same biological function. Using the similarity search option the module evaluates the degree of homology using BLASTp program [35]. Presence of targets from ∑ list in DrugBank, TTD, and database of enzyme targets for marketed drugs with same biological function acts as an evidence for their druggable property [54]. On the other hand, its absence indicates the novelty of the target and hence, classified as ‘novel target’ [55].

## Results and Discussion

The present study represents a new hierarchical *in silico* approach to identify and characterize potential therapeutic candidates in *M. abscessus* ATCC 19977, an emerging multi-drug resistant pathogen. Considering functionally important proteins of this pathogen from five different origins as input datasets, the hierarchy of analysis resulted in the identification of a list of efficient drug targets. In general, proteins of length less than 100 amino acids (mini proteins) are considered as insignificant and are excluded from the analysis [12], [56], [57]. Mini proteins are found to be most abundant across several prokaryotic genomes and they play a crucial role in various biological processes and regulatory functions [58]. Such mini proteins were also included in this study.

The current target identification and characterization approach comprises three phases of analyses (Figure 1). Phase I involves the mining of protein datasets through chokepoint, plasmid protein, pathway, virulence factor, and resistance genes and protein network analysis. Phase II passes the mined protein datasets through the subtractive channel of analysis. This comprises four sequence level analyses for filtering proteins that are non-homologous to human, essential for the survival of the pathogen, non-homologous to anti-targets, and non-homologous to gut microflora. The candidate proteins resulting from phase I and II form the final list of potential targets (∑) (Figure 2). The ∑ list of efficient targets is qualitatively characterized in phase III. This phase includes (A) identification of protein location in the bacterial cell, (B) detection of broad spectrum targets by homology search against a list of pathogens, (C) identification of interactome and its evaluation by node deletion process, (D) prediction of functionality by recognizing protein signatures, and (E) evaluation of druggability by similarity search against drug target databases (Figure 2). Various analyses performed and the numbers of proteins selected and excluded at different steps of the channel are given in Table 1.

**Table 1:** Analyses performed and datasets used in the target identification channel.

Phase	Analysis	Technique	Input	Passed	Failed
I	Chokepoint Analysis	(i) 1179 (P*-440, C#-426, S$-313) Mab	**762(α)**	**631(α1)**	**131(α1**′**)**
		against 880 Hsa Chokepoint reactions	P-294;C-284;S-184	P-241;C-234;S-156	P-53;C-50;S-28
		(ii) Presence of Chokepoint reactions in	**631(α1)**	**394(α2)**	**237(α2**′**)**
		KEGG	P-241;C-234;S-156	P-145;C-145;S-104	P-96;C-89;S-52
	Plasmid Protein Analysis	Sequence Retrieval	**21(β)**	−	−
	Pathway Analysis	(i) 110 Mab against 248 Hsa Pathways	**(γ)**	**1254(γ1)**	**(γ1**′**)**
		(ii) All proteins from distinct pathways &	Distinct-31	Proteins-498	Hsa & Mab
		unique proteins from Common pathways	Common-79	Proteins-756	common Proteins
	Virulence Factors	(i) Presence of virulence proteins in mab	**336(δ)**	**206(δ1)**	**130(δ1**′**)**
	Analysis	KEGG/NCBI	VFDB-222;MH-32	VFDB-92;MH@-32	VFDB-130;MH-0
			non-MH-82	non-MH-82	non-MH-0
		(ii) Absence of input proteins in Human	**206(δ1)**	**200(δ2)**	**6(δ2**′**)**
		pathways	VFDB-92;MH-32	VFDB-92;MH-30	VFDB-0;MH-2
			non-MH-82	non-MH-78	non-MH-4
	Resistance Genes and	Resistance causing and associated	**70(ε)**	**68(ε1)**	**2(ε1**′**)**
	Protein Network	proteins (STRING)	Proteins-4	Proteins-68	Proteins-2
	Analysis	Absence of input proteins in Human	Interactors-66	i)0; ii)2;	i)1; ii)0;
		pathways	i)1; ii)2;iii)13;iv)50	iii)13;iv)49	iii)0;iv)1
II	Non-Homology	1900 Mab proteins against nr *Homo*	**Input-1900**	**805(φ)**	**1095(φ**′**)**
	Analysis**(φ)**	*sapiens* database (BLASTp)	**α-**394(α2);**β**-21;	**α-**280;**β-**19;	**α-**114;**β -**2;
			**γ-**1254(γ1);	**γ-**374;	**γ-**880;
			**δ-**163(δ2);**ε-**68(ε1)	**δ-**118;**ε-**14	**δ-**45;**ε-**54
	Essentiality Analysis **(χ)**	805 Mab proteins against DEG	**Input-805**	**491(χ)**	**314(χ**′**)**
		(BLASTp)	**α-**280**;β-**19;	**α-**194;**β-**1;	**α-**86;**β-**18;
			**γ-**374;**δ-**118;**ε-1**4	**γ-**244;**δ-**38;**ε-**14	**γ-**130;**δ-**80;**ε-**0
	Anti-target Non-	303 Mab proteins against Anti-Targets in	**Input-303**	**262(ψ)**	**41(ψ**′**)**
	Homology Analysis **(ψ)**	Human (BLASTp)	**α-**81;**β-**1;	**α-**73;**β-**1;	**α-**8;**β-**0;
			**γ-**169;**δ-**38;**ε-**14	**γ-**144;**δ-**32;**ε-**12	**γ-**25;**δ-**6;**ε-**2
	Gut Flora Non-	273 Mab proteins against nr database of	**Input-262**	**41(ω)**	**221(ω**′**)**
	Homology Analysis **(ω)**	83 gut flora organisms (BLASTp)	**α-**73;**β-**1;	**α-**6;**β-**0;	**α-**67;**β-**1;
			**γ-**144;**δ-**32;**ε-**12	**γ-**22;**δ-**13;**ε-**0	**γ-**122**;δ-**19;**ε-**12
III	Cellular Localization	CELLO & PSORTb protein location	**40(∑)**		
	Analysis **(A)**	prediction			
	Broad Spectrum	Homology search against nr database of	**40(∑)**		
	Analysis **(B)**	240 pathogens			
	Interactome Analysis **(C)**	Interactors from STRING & Node	**40(∑)**		
		deletion process			
	Functionality	InterProScan function prediction	8 Hypothetical		
	Analysis **(D)**		proteins		
	Druggability	Targets in Drug Bank, TTD	**40(∑)**		
	Analysis **(E)**	and Marketed drugs database			

* Producing chokepoint reactions;

# Consuming chokepoint reactions;

$ Simultaneously producing & consuming chokepoint reactions;

@MH: Mycobacterial Homologs.

Mab: *Mycobacterium abscessus*; Hsa: *Homo sapiens*; KEGG: Kyoto Encyclopedia of Genes and Genomes database; VFDB: Virulence Factor Data Base; nr: non-redundant; DEG: Database of Essential Genes; TTD: Therapeutic Target Database.

### Phase I: Mining of Protein Datasets

**Chokepoint analysis.**

Chokepoint report of both host and pathogen was generated using Pathway Tools 14.5 [27]. Currently, Pathway/Genome Database (PGDB) describes 4991 genes from chromosome and plasmid of *M. abscessus.* Of 4991 genes, 4941 are protein coding genes and 50 are RNA genes. Pathway Tools consider both metabolic pathway and non-pathway reactions for generation of chokepoint report. The generated report enlisted chokepoint enzymes with EC number, reaction equation, and chokepoint metabolites from different categories like producing, consuming, and simultaneously producing and consuming chokepoint reactions. Of 1179 chokepoint enzymes of *M. abscessus*, 440 were from producing, 426 from consuming, and 313 from simultaneously producing and consuming chokepoint reactions. The number of chokepoint proteins selected and excluded from the various analyses is given in Table 1 and Table S3. The initial dataset (α list) of chokepoint proteins resulting from Pathway Tools was subjected to two steps of manual curation. First step was done to identify unique chokepoint enzymes of *M. abscessus* by comparing the chokepoint report of both host and pathogen. Chokepoint enzymes that are unique to *M. abscessus* were selected (631 enzymes-α1 list) and enzymes common to both host and pathogen were excluded (131-α1′ list). Second step was performed to select chokepoint proteins from α1 list, based on the presence of their corresponding enzymatic reactions in KEGG pathway database [28]. Of 631 CP enzymes, 394 enzymes present in KEGG alone were selected (α2 list) and the rest were excluded (237-α2′ list). The selected α2 list of enzymes was then passed through the subtractive channel of analysis (Phase II) (Table S3). Protein catalyzing chokepoint reaction is considered as a highly prioritized quality of a potential drug target which has also been highlighted by several investigators [24], [26], [59].

#### Plasmid protein analysis

Protein sequences corresponding to 21 plasmid genes of *M. abscessus* were retrieved from NCBI-Nucleotide database [29] (Table 1 and Table S4). Most of the drug target identification approaches [12], [56], [57] consider protein sequences encoded by chromosomal genes alone as input. Here plasmid proteins were also included in the analysis, as they play an important role in adaptability of the pathogen to adverse environment. For instance, mercury resistance plasmid genes are believed to confer resistance to organomercury compounds [6]. Gene *merB* within *mer* operon facilitates detoxification of mercurial compounds, thereby helping the pathogen to tolerate mercury-mediated toxicity [60]. Hence, inhibition of such plasmid proteins can block the detoxification mechanism in pathogen inducing adverse effects through constant exposure and accumulation of organomercurial compounds [60]. In the present study, proteins encoded by plasmid genes were passed through the subtractive channel of analysis (Figure 1).

#### Pathway analysis

*In silico* comparative analysis of *H. sapiens* and *M. abscessus* pathways (γ list) related to various metabolic processes was performed. *M. abscessus* possesses 110 pathways, which are classified as metabolism (metabolism: carbohydrate, energy, lipid, nucleotide, amino acid, cofactors, vitamins, terpenoids, polyketides; biosynthesis and metabolism: glycan and secondary metabolites; biodegradation and metabolism: xenobiotics), genetic information processing (transcription, translation, folding, sorting and degradation, replication and repair), environmental information processing (membrane transport, signal transduction), and human diseases (infectious diseases). Pathways that are uniquely present in *M. abscessus* and absent in *H. sapiens* (248 pathways) were termed as distinct pathways, whereas pathways present in both host and pathogen were referred as common pathways. Of 110 pathways, 31 were found as distinct pathways and 79 as common pathways. Sequences of 498 proteins from distinct pathways and 756 unique proteins from common pathways were collected (γ1 list) and passed through the subtractive channel of analysis (Phase II) (Table 1 and Table S5). Most of the existing drug compounds act by frequently targeting proteins from cell wall, DNA, and protein biosynthesis [61]. Currently reported target identification and drug discovery approaches are narrowed down to lipopolysaccharide and peptidoglycan biosynthesis pathways as they play a vital role in bacterial cell protection [22], [62]. This restricted approach of targeting some specific pathways may be a large contributor for the development of multi-drug resistance among pathogenic bacteria [63]. The present methodology considers all functional pathways, including DOXP pathway, folate and fatty acid biosynthesis, protein secretion, and two-component system [61], which could pave new directions in anti-microbial drug discovery. In most of the bacteria, including the organism under study, synthesis of isopentenyl pyro phosphate (IPP), an intermediate in terpenoid backbone biosynthesis does not take place by the classical mevalonate pathway [64]. Alternatively, synthesis is done by MEP/DOXP, a non-mevlonate pathway [65]. Inhibition of DOXP pathway makes the cells swollen and also hinders the biosynthesis of both carotenoids and menaquinones [66], [67]. In bacteria, fatty acid biosynthesis is carried out by type II FAS, whereas in human by type I FAS pathways [68]. But *Mycobacteria* synthesize straight chain fatty acids by type I FAS and its elongation (mycolic acid, which makes mycobacterium more pathogenic and infectious) by type II FAS [69], [70]. Unlike mammals, microbes lack the carrier mediated active transporter system for the uptake of pre-formed folate. Hence, most of the bacteria must undergo *de novo* biosynthesis of folate [71]. Proteins involved in folate biosynthesis are efficient drug targets as its derivatives are involved in nucleotide and amino acid biosynthesis [72]–[74]. Using two component signal transduction system the organism senses the changes in the environment, interprets the signals, and modifies the expression of some genes as response, which facilitates its survival [63]. Moreover, this system is also found to be associated with the pathogenicity of the organisms and its absence in human makes them attractive drug targets [75], [76]. Hence, inhibition of proteins involved in two component system could reduce virulence of the organism and hinder its adaptability to the changing environmental conditions.

#### Virulence factors analysis

Comparison of the pathogenomic composition of 24 mycobacterial genomes is reported in VFDB [31]. Chromosome and plasmid encoded virulence factors from 12 different categories, including amino acid and purine metabolism, cell surface components, lipid and fatty acid metabolism, secreted proteins, and stress adaptation are enlisted in VFDB [31]. In addition to 222 virulence factors of genus *Mycobacterium* from VFDB, 32 mycobacterial and 82 non-mycobacterial virulence factors, which are orthologous to *M. abscessus* reported in literature [6] were also collected, resulting in a list of 336 virulence factors (δ) (Table 1 and Table S6). Of 336, 206 (δ1 list) virulence proteins present in *M. abscessus* were identified and downloaded from KEGG/NCBI database. The 206 (δ1 list) proteins were further short-listed based on their presence in KEGG human pathways. Virulence proteins absent in human pathways alone were selected (200-δ2 list) and further graded through the phase II analysis.

#### Resistance genes and protein network analysis

Four genes, namely, *erm*(41), *inhA*, *katG*, and *rpsL* responsible for resistance towards macrolide-based chemotherapy, isoniazid, and streptomycin in mycobacterium species were considered for this analysis [10], [33]. Protein sequences orthologous to these resistance causing proteins in *M. abscessus* were retrieved from NCBI. In addition, proteins functionally associated with these resistance causing proteins in *M. abscessus* were identified using STRING [34]. Proteins encoded by *erm*(41), *inhA*, *katG*, and *rpsL* genes had 1, 2, 13, and 50 interactors, respectively in their corresponding protein network. In general, resistance causing proteins and their associated proteins are considered as ‘potential drug targets’ [14], as inhibition of such proteins may block the drug resistance mechanism. Among 70 (ε list) resistance causing proteins and interactors, proteins that are absent in host metabolic pathways were selected (68-ε1 list) and carried to the next subtractive channel of analysis (Phase II) (Table 1 and Table S7).

### Phase II: Subtractive Channel of Analysis

Protein dataset resulted from phase I was screened based on the criteria of selectivity/specificity and essentiality (Figure 1). The selectivity/specificity of the proteins was determined by finding out proteins that are non-homologous to human proteome, anti-targets, and gut microflora proteome and the essentiality of the proteins was evaluated by a homology search against a known set of essential proteins from other bacteria.

#### Non-homology analysis

Consideration of proteins homologous to host as drug targets could adversely affect the host metabolism. Thereby, filtering proteins homologous to human proteome is considered as the first step in several *in silico* drug target identification approaches [18], [22]. Short-listed input datasets from chokepoint, plasmid, pathway, virulence factors, and resistance genes and protein network analysis were subjected to non-homology analysis. A total of 1900 *M. abscessus* proteins prioritized in phase I were subjected to homology search against whole proteome of *H. sapiens* (host) using BLASTp [35] with e-value threshold of 0.005. Among 1900 proteins, 394 proteins were resulted (α2 list) from chokepoint analysis, 21 from plasmid protein analysis, 1254 (γ1 list) from pathway analysis, 163 (δ2 list) from virulence factors analysis, and 68 (ε1 list) from resistance genes and protein network analysis. Proteins without any hits for the above mentioned e-value threshold were regarded as non-homologs, whereas those exhibiting hits were considered as close homologs. Out of 1900 input proteins (Table 1 and Table S3, S4, S5, S6, S7), 805 that are non-homologous to human (φ list: α-280, β-19, γ-374, δ-118, ε-14) were selected and 1095 homologous proteins (φ′ list: α-114, β-2, γ-880, δ-45, ε-54) were excluded from the analysis.

#### Essentiality analysis

The non-homologous protein (φ) list was further screened based on essentiality using DEG server with an expect value of 0.0001. The most crucial criterion of a potential drug target is that a target must be an indispensable protein for the survival of the pathogen [22]. Of 805 input proteins identified as non-homologous to human, 491 proteins (χ list: α-194, β-1, γ-244, δ-38, ε-14) that have homologs with less than the given threshold value were regarded as essential for the survival of the pathogen and selected for the successive analyses. Proteins not showing any hits (χ′ list: α-86, β-18, γ-130, δ-80, ε-0) against DEG were considered as non-essential and excluded from the analysis. The redundant proteins in χ list resulted from pathway and chokepoint analysis were removed and the final list consisting of 303 proteins (χ list: α-81, β-1, γ-169, δ-38, ε-14) was then passed through anti-target non-homology analysis (Table 1 and Table S3, S4, S5, S6, S7).

#### Anti-target non-homology analysis

For avoiding severe toxic effects in host, identification of proteins non-homologous to anti-targets (human essential proteins) is considered as a critical step in this study. Each of the χ list pathogen proteins was subjected to homology search against a list of 216 anti-targets (Text S1) using BLASTp [35]. E-value threshold was set to 0.005 and proteins exhibiting hits below this threshold were regarded as homologs. Of 303 proteins in χ list (Table 1 and Table S3, S4, S5, S6, S7), 262 were non-homologous (ψ list: α-73, β-1, γ-144, δ-32, ε-12) and 41 (ψ′ list: α-8, β-0, γ-25, δ-6, ε-2) were close homologs to anti-targets. Proteins in ψ′ list were eliminated, as drugs inhibiting such targets may interfere in host metabolism causing severe adverse effects [14]. During 1960–1999, several drugs from different categories like non-steroidal anti-inflammatory drugs, non-narcotic analgesics, antidepressants, and vasodilators were withdrawn [77]. These drugs posed various health related complications causing hepatic, hematologic, cardiovascular, and dermatologic toxicities and carcinogenic effects [77]. Trovafloxacin (1999) [78], bromfenac (1998) [79], and ebrotidine [80] were withdrawn from worldwide pharmaceutical markets due to their hepatotoxic effects, whereas benoxaprofen (1982) [81] and fenclofenac (1984) [82] were withdrawn as a result of carcinogenic effects. The usage of sulfacarbamide (1988) [83] and sulfamethoxypyridazine (1986) [84] were banned due to their dermatologic and hematologic reactions in host. Encainide (1991) [82] and terfenadine (1997) [85] were withdrawn due to the safety concerned with cardiovascular reactions [77]. Troglitazone, cerivastatin, and rofecoxib were also withdrawn in the early 21^st^ century owing to the increased incidence of serious adverse outcomes [86].

#### Gut flora non-homology analysis

The resulting ψ list of proteins that are non-homologous to host and essential were subjected to BLASTp search [35] against the whole proteome of 83 gut microflora (Text S2) with e-value threshold of 0.0001. The co-evolved microflora in human gut helps in assimilation of poorly digestible dietary component, vitamin synthesis, and degradation of xenobiotics [87]–[91]. The gut prokaryotic symbionts also play a key role in human health by providing resistance to colonization of pathogens and opportunistic bacteria [89], [92]. Hence, deterioration of gut microflora population may result in lack of a first line of defense against pathogenic invasion and also lead to nutrition insufficiency in host [89]. Proteins showing less than 10 homologs with e-value threshold below 0.0001 were identified and selected for the further qualitative analyses. From 262 proteins given as input (Table 1 and Table S3, S4, S5, S6, S7), 41 proteins (ω list: α-6, β-0, γ-22, δ-13, ε-0) were selected and 221 proteins (ω′ list: α-67, β-1, γ-122, δ-19, ε-12) showing more hits (>10) were excluded. Proteins meeting all the selection criteria assigned in phase II were regarded as potential drug targets in *M. abscessus*.

The identified potential targets (ω list) resulted from phase I and II were combined and a final list of drug targets ∑ was formed (Table 1). Among the targets in ω list, MAB_2249 was short-listed from both pathway and virulence factors analysis. The redundancy was removed to generate a list of non-redundant potential drug targets (∑ list). This resulted in 40 promising targets which satisfy the essential criteria of an ideal target (Table 2). The final list of targets consists of proteins involved in amino acid, energy, carbohydrate, and lipid metabolism; degradation of xenobiotics; genetic information processing; signal transduction; and virulence. Virulence factors and pathway proteins involved in amino acid and fatty acid metabolism provide major contribution to the final list of potential targets. Proteins participating in multiple pathways are believed to be more efficient drug target candidates, as inhibiting the activity of such targets could increase lethal effect by obstructing the function of several metabolic processes of the pathogen. Out of 40 ∑ list targets, 12 targets, namely, MAB_3673, MAB_2754, MAB_2423, MAB_2424, MAB_2425, MAB_0754c, MAB_1237, MAB_2157, MAB_2550c, MAB_3354, MAB_4403c, and MAB_2822c were found to participate in more than one metabolic pathway.

**Table 2:** List of promising therapeutic targets in *M. abscessus*.

Target No	KEGG ID	Gene Product Definition	Length(aa)	Associated Pathway	UniProt ID	Choke point Protein	Virulence Factor	*COG ID
1	MAB_0295	putative phenazine	393	mab00400	B1MFJ4	YES	NO	COG3200E
		biosynthesis protein						
		PhzC						
2	MAB_1987	3-deoxy-D-arabino-	462	mab00400	B1MP18	YES	NO	COG3200E
		heptulosonate 7-						
		phosphate synthase						
		AroG						
3	MAB_0054c	cyanate hydratase	156	mab00910	CYNS	YES	NO	COG1513P
4	MAB_1028c	cyanate hydratase	146	mab00910	B1MJG2	YES	NO	COG1513P
5	MAB_3072	N-acetylglutamate	186	mab00330	B1MD29	YES	NO	COG1246E
		synthase						
6	MAB_3310c	oxygen-insensitive	215	mab00633	B1MDR7	YES	NO	COG0778C
		NAD(P)H						
		nitroreductase						
7	MAB_2249	lysine-N-oxygenase	429	mab01053	B1MAQ9	NO	YES	−
		MbtG						
8	MAB_3673	succinate	140	mab00020	B1MG02	NO	NO	COG2009C
		dehydrogenase		mab00190				
		(cytochrome b-556		mab00623				
		subunit) SdhC		mab00650				
9	MAB_2754	hypothetical protein	328	mab00190	B1MC62	NO	NO	COG1612O
				mab00860				
				mab02020				
10	MAB_2423	urease subunit	100	mab00230	URE3	NO	NO	COG0831E
		gamma UreA		mab00330				
				mab00791				
11	MAB_2424	urease subunit beta	110	mab00230	B1MB84	NO	NO	COG0832E
		UreB		mab00330				
				mab00791				
12	MAB_2425	urease subunit alpha	577	mab00230	URE1	NO	NO	COG0804E
				mab00330				
				mab00791				
13	MAB_2917	putative hydrolase	348	mab00650	B1MCM4	NO	NO	COG1870I
14	MAB_0754c	fatty acid desaturase	335	mab00061	B1MI28	NO	NO	−
				mab01040				
15	MAB_1237	acyl-ACP desaturase	268	mab00061	B1MKN3	NO	NO	−
		DesA2		mab01040				
16	MAB_2157	acyl-[acyl-carrier	306	mab00061	B1MAG7	NO	NO	−
		protein] desaturase		mab01040				
17	MAB_2550c	fatty acid desaturase	327	mab00061	B1MBL0	NO	NO	−
				mab01040				
18	MAB_3354	acyl-[acyl-carrier	328	mab00061	B1MDW1	NO	NO	−
		protein] desaturase		mab01040				
		DesA1						
19	MAB_4403c	acyl-[acyl-carrier	322	mab00061	B1MJW6	NO	NO	−
		protein] desaturase		mab01040				
20	MAB_4145	alpha,alpha-	503	mab00500	B1MIJ7	NO	NO	COG0380G
		trehalose-phosphate						
		synthase						
21	MAB_1704c	1-aminocyclopropane-	340	mab00640	B1MN77	NO	NO	COG2515E
		1-carboxylate						
		deaminase						
22	MAB_1335c	transferase	319	mab00300	B1MLJ6	NO	NO	COG2171E
23	MAB_0086	putative taurine	310	mab00430	B1MEC2	NO	NO	COG2175Q
		dioxygenase						
24	MAB_4209c	putative	316	mab00430	B1MIR1	NO	NO	COG2175Q
		dioxygenase						
25	MAB_2173	proteasome (subunit	260	mab03050	PSA	NO	NO	COG0638O
		alpha) PrcA						
26	MAB_2878c	hypothetical protein	558	mab02010	B1MCI6	NO	NO	COG0747EP
27	MAB_0400c	exodeoxy	1077	mab03440	B1MFU9	NO	NO	COG1330L
		ribonuclease V						
		subunit gamma						
28	MAB_2822c	DNA-directed RNA	102	mab00230	B1MCD0	NO	NO	COG1758K
		polymerase subunit		mab00240				
		omega		mab03020				
29	MAB_3125c	hypothetical protein	202	−	B1MD81	NO	YES	COG1670J
30	MAB_2233c	hypothetical protein	521	−	B1MAP3	NO	YES	−
31	MAB_0046	PE family protein	102	−	B1MAP1	NO	YES	−
32	MAB_2227c	hypothetical protein	287	−	B1MAN7	NO	YES	−
33	MAB_2229c	PE family protein	97	−	B1MAN9	NO	YES	−
34	MAB_3757	hypothetical protein	511	−	B1MG86	NO	YES	−
35	MAB_3759c	hypothetical protein	493	−	B1MG88	NO	YES	−
36	MAB_0852	polyketide synthase	463	−	B1MIC4	NO	YES	−
		associated protein						
		PapA2						
37	MAB_0297	isochorismatase	211	−	B1MFJ6	NO	YES	COG1535Q
		(phenazine						
		biosynthesis) PhzD						
38	MAB_0908	putative	305	mab00360	B1MJ46	NO	YES	COG3396S
		phenylacetic acid						
		degradation protein						
		PaaC/phenylacetate-						
		CoA oxygenase,						
		PaaI subunit						
39	MAB_2027	putative acyl carrier	160	−	B1MP58	NO	YES	COG0236IQ
		protein						
40	MAB_2286	hypothetical protein	328	−	B1MAU6	NO	YES	COG2898S

COG: Cluster of Orthologous Group;

* Functional categories of COGs: E-Amino acid transport and metabolism; P-Inorganic ion transport and metabolism; C-Energy production and conversion; O-Posttranslational modification, protein turnover, chaperones; I-Lipid metabolism; G-Carbohydrate transport and metabolism; Q-Secondary metabolites biosynthesis, transport and catabolism; L-DNA Replication, recombination, and repair; K-Transcription; J-Translation, ribosomal structure and biogenesis; S-Function unknown.

### PHASE III: Qualitative Characterization of the Short-listed Targets

In this phase, the therapeutic targets in ∑ list were further explored through various qualitative analyses, namely, cellular localization (A), broad spectrum (B), interactome (C), functionality (D), and druggability analysis (E) (Figure 2).

#### Cellular localization analysis (A)

In order to characterize the short-listed targets as drug and vaccine targets, their distribution within the bacterial cell was determined using two prediction servers PSORTb and CELLO. Target proteins located in cytoplasm can be used as drug targets, whereas extracellular and membrane bound proteins can act as vaccine targets [12], [93]. Based on the localization score, PSORTb sorted the location of 23 targets as cytoplasmic and 4 as cytoplasmic membrane. The location of 12 proteins was predicted to be unknown since more than one site score identical minimal value. MAB_0400c was predicted to have multiple localization sites as it showed good score for both cytoplasmic and cytoplasmic membrane. The subcellular localization of the identified targets, predicted by PSORTb, was cross-checked by using CELLO in which the location of 31 targets was evaluated as cytoplasmic, 6 as membrane, and 3 as extracellular, based on the reliability score. CELLO predicted the location of 12 targets (characterized as unknown by PSORTb) as cytoplasmic (9) and extracellular (3). Although PSORTb predicted MAB_2917 as a cytoplasmic protein, CELLO predicted it as a membrane protein (Table 3). Target proteins involved in membrane transport pathway and virulence of the pathogen were found to be extracellular. Location of 31 targets was predicted as cytoplasmic and 8 targets as cytoplasmic membrane/extracellular (Table 3) which could possibly serve as drug and vaccine targets, respectively.

**Table 3:** Qualitative characterization of ∑ list targets.

Target No	KEGG ID	Subcellular Localization	Broad Spectrum Property	Interactors	Function Prediction	Druggability
1	MAB_0295	Cytoplasmic	No(52)	6	Not required	Novel
2	MAB_1987	Cytoplasmic	No (51)	4	Not required	Novel
3	MAB_0054c	Cytoplasmic	No (37)	8	Not required	Novel
4	MAB_1028c	Cytoplasmic	No (36)	6	Not required	Novel
5	MAB_3072	Cytoplasmic	Yes (151)	17	Not required	Novel
6	MAB_3310c	Cytoplasmic	Yes (130)	2	Not required	Druggable
7	MAB_2249	Cytoplasmic	No (49)	4	Not required	Novel
8	MAB_3673	Membrane	Yes (115)	18	Not required	Novel
9	MAB_2754	Membrane	No (79)	4	Heme A synthase	Novel
10	MAB_2423	Cytoplasmic	No (93)	7	Not required	Druggable
11	MAB_2424	Cytoplasmic	No(95)	7	Not required	Druggable
12	MAB_2425	Cytoplasmic	Yes (139)	7	Not required	Druggable
13	MAB_2917	Cytoplasmic	No (78)	1	Not required	Novel
		/Membrane				
14	MAB_0754c	Cytoplasmic	No (29)	5	Not required	Novel
15	MAB_1237	Cytoplasmic	No (58)	4	Not required	Novel
16	MAB_2157	Cytoplasmic	No (35)	4	Not required	Novel
17	MAB_2550c	Cytoplasmic	No (31)	4	Not required	Novel
18	MAB_3354	Cytoplasmic	No (37)	4	Not required	Novel
19	MAB_4403c	Cytoplasmic	No (26)	4	Not required	Novel
20	MAB_4145	Cytoplasmic	No (62)	6	Not required	Druggable
21	MAB_1704c	Cytoplasmic	Yes (122)	2	Not required	Druggable
22	MAB_1335c	Cytoplasmic	Yes (127)	4	Not required	Novel
23	MAB_0086	Cytoplasmic	No (72)	2	Not required	Druggable
24	MAB_4209c	Cytoplasmic	No (70)	2	Not required	Druggable
25	MAB_2173	Cytoplasmic	No (23)	9	Not required	Druggable
26	MAB_2878c	Extracellular	Yes (109)	10	Bacterial extracellular	Novel
					solute- binding protein	
27	MAB_0400c	Cytoplasmic	Yes (118)	2	Not required	Novel
28	MAB_2822c	Cytoplasmic	Yes (103)	43	Not required	Druggable
29	MAB_3125c	Cytoplasmic	No (60)	3	Acyl-CoA N-	Novel
					acyltransferase,	
					Siderophore	
					biosynthesis protein	
30	MAB_2233c	Membrane	No (25)	7	Type VII secretion	Novel
					system protein	
31	MAB_0046	Extracellular	No (47)	1	Not required	Novel
32	MAB_2227c	Cytoplasmic	No (16)	1	EspG family protein	Novel
33	MAB_2229c	Extracellular	No (33)	1	Not required	Novel
34	MAB_3757	Membrane	No (31)	3	Type VII secretion	Novel
					system, YukD- like	
					protein	
35	MAB_3759c	Membrane	No (43)	8	Type VII secretion	Novel
					system protein	
36	MAB_0852	Cytoplasmic	No (43)	7	Not required	Novel
37	MAB_0297	Cytoplasmic	Yes (125)	7	Not required	Novel
38	MAB_0908	Cytoplasmic	No (46)	8	Not required	Novel
39	MAB_2027	Cytoplasmic	Yes (164)	3	Not required	Novel
40	MAB_2286	Cytoplasmic	No (81)	6	Acyl-CoA N-	Novel
					acyltransferase,	
					lysylphosphotidyl	
					Glycerol synthetase	

#### Broad spectrum analysis (B)

Comparative sequence analysis of the screened targets with medically important mycobacterial species and other bacterial pathogens facilitates the evaluation of proposed targets acting as ideal broad spectrum target. BLASTp homology search [35] against whole proteome of each of the 240 bacterial pathogens (Text S3) resulted in the identification of possible broad spectrum target [14]. From the homology search, 11, 12, and 17 targets were found to have close homologs in more than 100, 50, and less than 50 (ranges from 15–49) pathogens, respectively (Table 3). The analysis also revealed that ∑ list proteins exhibit homology to the proteome of many medically important non-tuberculous mycobacterial pathogens, such as *M. avium* complex, *M. intracellulare*, *M. kansasii*, *M. marinum*, and *M. smegmatis.* Among ∑ list proteins, 11 targets, which showed homology to more than 100 pathogens, were considered as broad spectrum target candidates (Table 3). Drug molecules designed to inhibit such broad spectrum targets may facilitate in eradication of wide range of pathogens. Targets resulting from virulence factor analysis were observed to have less homologs in other pathogenic bacteria as most of the virulence proteins are often specific to the pathogen [94]. Five targets exhibiting less (<30) homology were regarded as ‘probable Mab specific target’ (Table 3). Such pathogen specific targets may reduce the risk of development of drug resistance in wide range of pathogens [14]. In addition, COG search also revealed that most of the ∑ list targets have homologs in other bacterial pathogens (Table 2).

#### Interactome analysis (C)

The analysis on protein interaction network was performed to study the functional importance of short-listed targets in the metabolic network. The high confidence protein network of each ∑ list target was constructed using STRING database [34]. The predicted functional interactors of the query target, the prediction method and its confidence score are tabulated in Table S1. MAB_3072 (chokepoint protein), MAB_3673, and MAB_2822c resulted from pathway analysis were found to have more than 10 interactors. The protein interactome of about 15 targets had more than five interactors (Table 3). Target protein with more interactors is considered as metabolically important active protein which can act as an appropriate drug target [26], [48]. The protein interaction network was subjected to Cytoscape analysis using which each interactome was analyzed by node deletion process [14]. In the network analysis, the duplicated edges were removed and the changes in the essential parameters like connected components, network density, clustering coefficient, characteristic path length, network centralization, and network heterogeneity on node deletion process are recorded in Table S2. From node deletion process, it was observed that query target node deletion resulted in drastic changes in the network parameters than any other node, showing its importance in the network. In networks of MAB_0295, MAB_1987, MAB_0054c, MAB_1028c, MAB_3072, MAB_3310c, MAB_2249, MAB_3673, MAB_0754c, MAB_4145, MAB_1335c, MAB_2173, MAB_2878c, MAB_3125c, MAB_2233c, MAB_3757, MAB_3759c, and MAB_0852, knocking out the target node led to disruption of paths and increased the number of connected components indicating its role in maintaining the integrity of the network. The importance of the query node was also confirmed by the significant decrease in the density and centralization of the network [95].

The average shortest path length, betweenness centrality, degree, neighborhood connectivity, and topological coefficient of every node in each target’s network are tabulated for node attributed analysis in Table S2. In edge attributed analysis, the interactors of each query node, its confidence value, edge weight, and betweenness are registered (Table S2). Less edge weight shows better connectivity between nodes and it was calculated by the 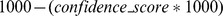 [46]. The significance of the target protein was observed by the critical change in the network associated parameters. This study along with the node and edge attributed parameter analysis proves the functional importance of the query node in the interactome.

#### Functionality analysis (D)

The hypothetical proteins in the ∑ list were analyzed using INTERPROSCAN in order to predict their function. Out of 40 final short-listed targets, eight proteins, namely, MAB_2754, MAB_2878c, MAB_3125c, MAB_2233c, MAB_2227c, MAB_3757, MAB_3759c, and MAB_2286 resulted from virulence factors and pathway analysis are hypothetical (Table 2). Hypothetical protein MAB_2754 from unique pathway analysis was predicted to be a Heme A synthase protein, which is reported to involve in heme A synthesis and oxidation-reduction process in *Bacillus subtilis* and *Rhodobacter sphaeroides* [96]. Hypothetical protein MAB_2878c (OppA) from common pathway analysis was predicted to be a bacterial extracellular solute-binding protein and involves in transporter activity. It has been reported that OppA protein from ABC transporter pathway of Gram-positive bacteria is included in protein Family 5 [97]. The remaining six hypothetical proteins, namely, MAB_3125c, MAB_2233c, MAB_2227c, MAB_3757, MAB_3759c, and MAB_2286 are factors responsible for the virulence of *M. abscessus*. The Interproscan result of MAB_3125c predicted it as an acyl-CoA N-acyltransferase enzyme and it also represents siderophore biosynthesis protein domain. The Interproscan result of the hypothetical proteins MAB_2233c and MAB_3759c envisaged it as type VII secretion system EccB proteins [98]. MAB_2227c is supposed to be a member of EspG family involved in the ESAT-6 secretion system and plays vital role in virulence and disease establishment in the host [99]. Interproscan of MAB_2286 protein recognized it as acyl-CoA N-acyltransferase and lysyl-phosphotidylglycerol synthetase enzyme. The protein MAB_3757 was predicted to be type VII secretion system EccD and YukD-like protein. In *Mycobacterium tuberculosis*, EccD proteins were found to be responsible for replication and macrophage suppression, thereby, playing a pivotal role in virulence [100], [101]. Thus, the functionality analysis of the hypothetical proteins in the ∑ list divulges their functional importance (Table 3).

#### Druggability analysis (E)

In the current approach, the druggability of the short-listed potential targets was evaluated by sequence similarity search against targets from DrugBank [50], marketed drug database [51], and TTD [53] (Table 3). BLASTp search [35] against DrugBank targets with FDA approved drugs, revealed that MAB_2425 is homologous to a known target (bacterial urease alpha subunit) with an e-value of 0. This known target has two approved inhibitors, namely, acetohydroxamic acid and ecabet. The urease alpha subunit is involved in vital pathways, such as amino acid metabolism, nucleotide metabolism, and xenobiotic degradation of *M. abscessus*. In addition, eight other short-listed targets, namely, MAB_3310c, MAB_2423, MAB_2424, MAB_4145, MAB_1704c, MAB_0086, MAB_4209c, and MAB_2173 have homology to DrugBank targets with experimental drugs. By a similar homology search against TTD, aligned targets with e-value less than 10^−5^ alone were considered as significant homologs. Two targets MAB_2425 and MAB_4145 showed similarity to TTD targets, designated as successful and research targets, respectively. EC number search in 13 bacterial targets of marketed drugs revealed two proteins having same biological function with that of ∑ list targets, namely, MAB_2425 (3.5.1.5) and MAB_2822c (2.7.7.6). It also reports marketed drugs, namely, acetohydroxamic acid [102] and rifapentine [103] targeting the enzymes 3.5.1.5 and 2.7.7.6, respectively. All the other targets showing no similarity with these drug target databases were differentiated as novel targets (Table 3), which should be further validated experimentally.

### Potentially Active Drugs through Target Identification Channel

The efficiency of various drugs administered for the treatment of *M. abscessus* infections is reported in literature [3], [104], [105]. It has been demonstrated that amikacin, cefoxitin, imipenem, azithromycin, tigecycline, moxifloxacin, gatifloxacin are the currently used active drugs [1], [7], although the disease isolates are not uniformly susceptible to these drugs [8]. The increasing prevalence of adverse effects associated with various antibiotic treatment procedures necessitates an improved target prioritization method. It is also evident that the side effects of drug compounds are further amplified with intense treatment regimens [106]. Amikacin, an aminoglycoside improving the survival of cystic fibrosis patients, causes major side effects like kidney injury, hearing impairment, and vestibular toxicity [106]. Cefoxitin, a cephalosporin imposes a deterioration of human colonial microbiota by reducing the population of anaerobic and enterobacteria, which ultimately leads to colonization of other pathogenic bacteria [107], [108]. Administration of imipenem (first carbapenem antibiotic) causes several side effects, including nausea, vomiting, and seizures in both adult and children under medication for various bacterial infections [109]–[111]. Resolvable abdominal pain, ENT infections, diarrhea, headache, and vomiting are the adverse effects frequently reported in cystic fibrosis patients associated with the azithromycin treatment regimens [112]. It has been demonstrated by analyzing the tolerability of tigecycline that this antibiotic poses a minor disturbance in the oropharyngeal microflora, which decrease the count of enterococci and *Escherichia coli* in the gut microflora leading to undesirable consequences [113]. The evaluation of moxifloxacin effects on human intestinal flora reported a significant increase in the population of *Candida* species, fungal pathogens [114]. Frequent adverse effects, such as nausea, dizziness, and diarrhea were also reported in some patients under moxifloxacin treatment regimens [115]. Gatifloxacin was recently withdrawn due to its adverse glycaemic effects and hepatotoxicity [116], [117]. Target proteins corresponding to these active drug compounds were identified from DrugBank database [50]. Out of these drug target proteins, fourteen targets were found to be encoded by *M. abscessus* genome. To evaluate the fitness of the targets of the above mentioned active drugs, target sequences were collected from NCBI database and passed through the four different steps of phase II (Table 4). Analysis revealed that all the targets of known active drugs failed one or more steps of the subtractive channel of analysis signifying the need for the identification of novel targets for the development of new drugs against *M. abscessus*.

**Table 4:** Targets of known active drugs through the subtractive channel of analysis.

			SUBTRACTIVE CHANNEL OF ANALYSIS			
Drugs	Targets	Pathway	Non-HomologyAnalysis	EssentialityAnalysis	Anti-targetNon-HomologyAnalysis	Gut FloraNon-HomologyAnalysis
Amikacin	30S ribosomal protein S12	mab03010	X	✓	✓	X
Cefoxitin	Penicillin-binding protein 4	mab00550	✓	✓	✓	X
	Penicillin-binding protein 1A	−	✓	✓	X	X
	Penicillin-binding protein 3	mab00550	✓	✓	✓	X
	Beta-lactamase	mab02020	✓	X	✓	X
Imipenem	Penicillin-binding protein 2	−	✓	✓	✓	X
	Penicillin-binding protein 1A	−	✓	✓	X	X
	Penicillin-binding protein 3	mab00550	✓	✓	✓	X
	Beta-lactamase	mab02020	✓	X	✓	X
Azithromycin	50S ribosomal protein L4	mab03010	X	✓	✓	X
	50S ribosomal protein L22	mab03010	✓	✓	✓	X
Tigecycline	30S ribosomal protein S9	mab03010	X	✓	✓	X
	30S Ribosomal protein S12	mab03010	X	✓	✓	X
	30S ribosomal protein S13	mab03010	X	✓	✓	X
	30S ribosomal protein S14	mab03010	X	✓	✓	X
	30S ribosomal protein S19	mab03010	X	✓	✓	X
Moxifloxacin	DNA gyrase subunit A	−	X	✓	X	X
Gatifloxacin	DNA gyrase subunit B	−	X	✓	✓	X
	DNA gyrase subunit A	−	X	✓	X	X

### Conclusions

Employing a novel hierarchical *in silico* approach the present study reports a list of potential drug target candidates with their qualitative characterization. Proteins from five datasets (phase I) satisfying the following criteria: essential for the pathogen’s survival and non-homologous to host proteome, human anti-targets, and gut flora proteome are considered as potential therapeutic candidates. Chokepoint proteins, pathway proteins, and virulence factors are the contributors to the list of drug targets, as other two datasets- plasmid proteins and resistance genes/resistance-associated proteins could not pass through the different stages of subtractive channel of analysis. Further characterization of the final list of target candidates leads to the identification of drug and vaccine targets, which can enter into the drugs and vaccine development pipelines. Some of the targets are widely represented in different bacterial pathogens, including several clinically important mycobacterial species such as *M. avium* complex, *M. intracellulare*, *M. kansasii*, *M. marinum*, and *M. smegmatis*, making them potential candidates for broad spectrum drug development. The characterization stage also elucidates the targets’ functional association with metabolically interacting proteins, evaluates their druggable property, and predicts the function of the hypothetical proteins present in the target list. It is expected that the results of the present study could facilitate to develop drugs/vaccines, which would target merely the pathogen’s system, without harming the physiology or biology of the host. The systematic *in silico* approach adopted here can be further utilized in drug target identification for other pathogens of clinical interest.

## Supporting Information

Table S1 **Interactome of 40 targets of M. abscessus constructed from STRING.**
(XLS)

Table S2 **Node deletion and comparison of network parameters using Cytoscape.**
(XLS)

Table S3 **Chokepoint enzymes through the hierarchical analysis.**
(XLS)

Table S4 **Plasmid proteins through the hierarchical analysis.**
(XLS)

Table S5 **KEGG pathway-proteins through the hierarchical analysis.**
(XLS)

Table S6 **Virulence Factors through the hierarchical analysis.**
(XLS)

Table S7 **Resistance genes and resistance-associated proteins through the hierarchical analysis.**
(XLS)

Text S1 **Anti-targets in Human proteome.** Description of the 210 anti-target sequences used in the analysis.
(TXT)

Text S2 **Human gut microbiota.** List of 83 organisms in gut flora of normal human.
(TXT)

Text S3 **Bacterial Pathogens.** List of 240 pathogenic bacteria used in Broad Spectrum analysis.
(TXT)
